# OctopuSV and TentacleSV: a one-stop toolkit for multi-sample, cross-platform structural variant comparison and analysis

**DOI:** 10.1093/bioinformatics/btaf599

**Published:** 2025-10-31

**Authors:** Qingxiang Guo, Yangyang Li, Ting-You Wang, Abhirami Ramakrishnan, Rendong Yang

**Affiliations:** Department of Urology, Northwestern University Feinberg School of Medicine, Chicago, IL 60611, United States; Department of Urology, Northwestern University Feinberg School of Medicine, Chicago, IL 60611, United States; Department of Urology, Northwestern University Feinberg School of Medicine, Chicago, IL 60611, United States; Department of Urology, Northwestern University Feinberg School of Medicine, Chicago, IL 60611, United States; Department of Urology, Northwestern University Feinberg School of Medicine, Chicago, IL 60611, United States; Robert H. Lurie Comprehensive Cancer Center, Northwestern University Feinberg School of Medicine, Chicago, IL 60611, United States

## Abstract

**Motivation:**

Structural variants (SVs) influence gene regulation, disease progression, and diagnostics, yet integrating SV calls across platforms remains difficult due to inconsistent annotations, limited merging flexibility, and fragmented workflows. Ambiguous breakend (BND) annotations, which comprise many variant calls, are often discarded or misclassified, hindering variant characterization. Existing tools lack advanced merging operations essential for precise identification of disease-specific or somatic variants across samples or patient groups. Additionally, current SV analysis pipelines require extensive manual intervention and complex parameter tuning, compromising reproducibility and scalability. Addressing these gaps is crucial for improving the accuracy, interpretability, and clinical utility of SV analyses.

**Results:**

We developed OctopuSV and TentacleSV to address these long-standing challenges in SV analysis. OctopuSV features a specialized BND correction module that converts ambiguous BND annotations into canonical SV types, recovering important variants that are often overlooked by existing tools. Additionally, it provides advanced set operations (difference, complement, custom-defined) that enable sophisticated variant filtering without programming expertise, critical for identifying tumor-specific SVs or variants unique to specific sample groups. TentacleSV completes our solution by automating the entire SV analysis process from raw sequencing data to high-confidence callsets, ensuring consistency and reproducibility across projects. Benchmarking across short-read and long-read platforms showed superior F1 score, complete SV type consistency compared to existing tools. Our framework enables experimental biologists and clinical researchers to perform sophisticated analyses ranging from cancer subtype-specific SV identification to multi-sample comparative studies without requiring specialized programming skills.

**Availability and implementation:**

All codes are available at https://github.com/ylab-hi/OctopuSV; https://github.com/ylab-hi/TentacleSV.

## 1 Introduction

Structural variants (SVs)—genomic alterations larger than 50 bp—significantly impact genomic architecture leading to gene regulation changes, dosage modification, and chromosomal rearrangements (Belyeu *et al.* 2018, Mahmoud *et al.* 2019, Cameron *et al.* 2021). These variants (deletions [DELs], insertions [INSs], inversions [INVs], duplications [DUPs], and translocations [TRAs]) are crucial in cancer genomics and rare disease research (Deardorff *et al.* 2007, Sedlazeck *et al.* 2018). The expansion of short-read and long-read sequencing technologies has created urgent need for computational frameworks that reliably analyze SVs across diverse biological contexts and platforms (Sedlazeck *et al.* 2018, Chaisson *et al.* 2019, Zhang *et al.* 2024).

The scientific community has developed various tools for SV detection and integration (Cameron *et al.* 2019). SURVIVOR (Sedlazeck *et al.* 2018) enables SV merging across multiple samples using positional overlap, while Jasmine (Kirsche *et al.* 2023) merges SVs across samples using network-based algorithms that refines breakpoint locations through clustering techniques. Sniffles supports population calling via Sniffles2’s population mode (Smolka *et al.* 2024), while DELLY enables cohort analysis through force-calling workflows (Rausch *et al.* 2012). SVmerge (Zook *et al.* 2020) and CombiSV (Dierckxsens *et al.* 2021) support population-level analyses by combining calls from multiple samples. Viola-SV (Sugita *et al.* 2022) provides manipulation and visualization of SV callsets with BND-to-breakpoint conversion for signature analysis and limited SV caller support, whereas general-purpose tools such as BCFtools (Danecek *et al.* 2021) and VCFtools (Danecek *et al.* 2011) primarily handle SNP/indel operations without specialized functions for complex SV merging. PanPop (Zheng *et al.* 2024) implements sequence-aware merging strategies at population scale.

Despite these advances, critical challenges remain in SV analysis for applications in precision medicine and genomic studies (Gong *et al.* 2021). First, widely used SV detection tools—including Manta (Chen *et al.* 2016), LUMPY (Layer *et al.* 2014), and SvABA (Wala *et al.* 2018)—frequently report breakend (BND) annotations (Cameron *et al.* 2021), which describe breakpoints without specifying the precise SV type. Without proper handling, BND events are often discarded (Kosugi *et al.* 2019) or misclassified, potentially omitting biologically and clinically relevant variants (Barbitoff *et al.* 2024). Different SV callers may use inconsistent conventions (e.g. INV versus BND), complicating merging (Schikora-Tamarit and Gabaldón 2022). Second, existing merging tools support only basic operations (union, intersection), inadequate for modern research requiring identification of tumor- or subtype-specific SVs—operations currently requiring custom scripting (Sarwal *et al.* 2024). Third, SV analysis pipelines remain highly fragmented, demanding management of multiple softwares and complex configurations, compromising reproducibility and scalability for multi-sample projects (Belyeu *et al.* 2018). These barriers limit access for users lacking bioinformatics support.

To address these challenges, we developed OctopuSV, a versatile toolkit integrating variant calls from multiple detection methods and sequencing platforms, and TentacleSV, an automated pipeline providing end-to-end workflow from raw data to high-confidence calls. OctopuSV addresses the critical BND standardization while providing flexible merging capabilities. First, it features a specialized BND correction module that systematically converts ambiguous BND annotations into canonical SV types (DEL, INV, DUP, TRA), recovering important variants and enhancing merging accuracy across diverse datasets. Second, OctopuSV provides advanced set operations, (difference, complement, custom-defined) enabling practical variant filtering strategies without manual scripting. Third, TentacleSV automates the entire SV analysis process with minimal intervention, ensuring consistency across projects. Additional features include benchmarking and visualization (e.g. SV type distributions, chromosome maps), facilitating interpretation. These integrated capabilities support applications from cancer subtype-specific SV detection to multi-sample analysis (Rausch *et al.* 2012).

## 2 Materials and methods

### 2.1 Software overview and implementation

OctopuSV was implemented in Python and deployed through PyPI for streamlined installation. The software provides a command-line interface with subcommands for BND correction (“correct”), SV merging (“merge”), and benchmarking (“benchmark”), operating on customized VCF formats (Danecek *et al.* 2011) to ensure compatibility with existing workflows. The architecture comprises three layers: (i) input processing for diverse VCF formats, (ii) correction module for standardizing breakend representations, and (iii) merging module for integrating multi-caller or multi-sample outputs through flexible set operations. The name OctopuSV reflects the toolkit’s multi-armed modular design, enabling simultaneous handling of SV data from diverse sources and platforms. The name is unrelated to the small-variant caller Octopus (Cooke *et al.* 2021).

TentacleSV, a Snakemake-based automation layer, coordinates the complete pipeline from raw data through mapping, variant calling, and OctopuSV operations. Users can customize analysis through a configuration file (config.yaml) that specifies input locations, desired SV callers, and merging parameters. TentacleSV ensures reproducibility by consolidating all steps into one automated workflow.

### 2.2 BND correction algorithm

OctopuSV uses a hierarchical classification system converting BNDs into canonical SV types: DEL, INV, DUP, TRA, while preserving unresolved BNDs to respect underlying caller uncertainty. The algorithm classifies SVs into BND or non-BND events (Fig. 3c), with BNDs divided into same-chromosome and different-chromosome types. Same-chromosome BND pairs are categorized through bracket orientation patterns as DELs, INVs, DUPs, or retained as BND pairs for complex cases. Different-chromosome BNDs undergo mate-pair identification using positional coordinates with configurable tolerance (default: 3 bp), classifying them as balanced translocations (reciprocal exchanges with no net genetic material change), unbalanced translocations (transfers with genetic material gain or loss), or merging translocations (paired BND events representing single events). Events lacking mate pairs are classified as special no-mate pairs or labeled as unbalanced TRAs. We do not convert BNDs to INS as accurate insertion calling requires sequence content information that cannot be reliably inferred from breakpoint coordinates alone. BNDs lacking clear orientation or pairing support are retained to reflect possible complexity or ambiguity in the original call. Detailed conversion rules are provided in Dataset 1, available as supplementary data at *Bioinformatics* online.

### 2.3 SV merging and set operations

Following BND correction, OctopuSV merges SV calls from multiple callers by first sorting variants by SV type and organizing them by chromosome pairs. The merging approach differs between TRA and non-TRA events. For non-TRA variants (DEL, DUP, INV, INS), the algorithm groups events based on genomic positions using distance thresholds (50–150 bp), size similarity (ratio ≤ 1.3) and overlap (Jaccard index ≥ 0.7). For TRA events, more flexible position matching criteria are applied while ensuring consistent strand orientations between events.

OctopuSV provides various set operations for integrating results from multiple callers, including basic operations like union (keeping all unique variants), intersection (retaining only variants found by all callers), and threshold-based filtering (variants supported by a minimum number of callers, e.g. ≥2 or ≥3). Advanced operations unique to OctopuSV include maximum support thresholds (e.g. variants supported by ≤2 callers), custom set operations using logical operators, e.g. [(Manta AND SVABA) NOT (Lumpy OR Delly)], and single-caller extractions for analyzing results from specific tools. These flexible operations provide precise control over variant selection in the final output. OctopuSV also supports quality-based filtering using variant-level attributes (QUAL, supporting reads, depth, genotype quality) as post-processing steps, allowing users to apply dataset-specific quality thresholds. When multiple callers provide conflicting genotype calls for the same variant, OctopuSV uses a hierarchical resolution process: first by majority voting among callers, then by selecting the genotype with highest variant supporting read count (AD field), and finally by caller input file order when other criteria are equal.

### 2.4 Built-in benchmarking

OctopuSV includes a benchmarking module for evaluating SV calls against truth sets, following Genome in a Bottle (GIAB) consortium guidelines (Zook *et al.* 2020) with modified parameters. It implements a matching algorithm considering position-based matching (500 bp threshold), size similarity (minimum 0.7 ratio), and variant type matching. These thresholds, which have been widely validated in large-scale benchmarking studies (English *et al.* 2022), can be adjusted by users. The module calculates precision, recall, and F1 scores, globally and by SV type. Results are output as VCF files (TP, FP, FN) with JSON statistics.

### 2.5 Data preparation

Simulated and real datasets were prepared to assess OctopuSV’s performance across SV types and sequencing platforms. For simulated data, VISOR (v1.1.2) (Bolognini *et al.* 2020) embedded known SVs into GRCh38. Two distinct simulation strategies were used: (i) Real SV-based simulation sourced from dbVAR callsets, including CHM1 (nstd137) (Chaisson *et al.* 2015) and KWS1 (nstd106) (Huddleston *et al.* 2017), comprising 6167 DELs, 9899 INSs, 44 INVs, 3712 DUPs, and 380 TRAs; (ii) Balanced complex SV simulation with 15 000 canonical SVs (3000 each type) plus 3000 complex SVs created through a two-round VISOR HAck approach where overlapping variants (DEL-INV, DUP-INV, INV-DUP, DUP-DEL, INS-DEL combinations, 600 each) were embedded onto pre-existing simple SVs to simulate complex rearrangements. Variants were integrated into two haplotypes using VISOR HAck with default parameters, mimicking a 30:70 ratio of homozygous to heterozygous events, followed by in silico sequencing at 30× coverage for short-read NGS, Oxford Nanopore (ONT), and PacBio. Ground truth was constructed by merging variants from CHM1 and KWS1 dbVAR datasets, pre-processed through a rigorous pipeline: VCF files were sorted using BCFtools sort (v1.17) (Danecek *et al.* 2021), normalized against GRCh38.p13 using BCFtools norm, and merged with BCFtools concat, removing duplicates (-d both option).

For real data evaluation, NA12878 (HG001) (Zook *et al.* 2019) genome was analyzed using NIST NA12878 HG001 HiSeq 30× data and PacBio Sequel II CCS data with a mean read length of approximately 11 kb. Ground truth combined SVs from the 1000 Genomes Project (Sudmant *et al.* 2015) and long-read assembly-based NA12878 SV dataset (Kosugi *et al.* 2019). The final NA12878 ground truth dataset contained 49 754 DELs, 13 837 INSs, 8624 DUPs, and 1072 INVs, totaling 73 287 SVs. The same pre-processing pipeline was applied.

### 2.6 Genome mapping and SV calling

Short-read data were aligned to GRCh38 using BWA-MEM2 (v2.2.1) (Vasimuddin *et al.* 2019) with parameters “-t 12 -M -Y” and appropriate read group information. Long-read data were processed using minimap2 (v2.28-r1209) (Li 2018) with parameters “-ax map-ont” for ONT and “-ax map-hifi” for PacBio, supplemented by “--MD -t 8 -Y” and read group tags. Multiple SV callers were used: Manta (v1.6.0), LUMPY (v0.2.13), SvABA (v1.1.0), and DELLY (v1.3.1) for short-read data; CuteSV (v2.1.1) with parameters “-l 50 -L 5000000 -r 1000 -q 20 -s 3”, SVIM (v2.0.0) (Heller and Vingron 2019), PBSV (v2.10.0) (https://github.com/PacificBiosciences/pbsv), Sniffles (v2.5) (Sedlazeck *et al.* 2018), SVDSS (v2.0.0) (Denti *et al.* 2023), and DeBreak (Chen *et al.* 2023) with parameters “--min_support 2 -t 8 --rescue_large_ins --rescue_dup --poa” for long-read datasets.

### 2.7 BND prevalence analysis and correction evaluation

To assess BND correction importance, we performed two analyses across six datasets (NA12878 NGS/PacBio, VISOR NGS/ONT/PacBio, VISOR Complex NGS). First, we quantified BND prevalence as the proportion of BND-labeled events among all SVs reported by each caller. Second, we evaluated biological relevance by determining BND overlap with high-confidence reference variants.

To evaluate correction accuracy, BND entries were extracted and corrected using OctopuSV’s “correct” subcommand with a 3 bp position tolerance. Corrected events were then compared to ground truth sets (VISOR or NA12878) using matching thresholds of 50 bp (non-TRA) and 5000 bp (TRA). Type accuracy was calculated as the proportion of matched BNDs correctly converted to true SV types, and combined accuracy required both correct type and size concordance (ratio ≥0.7). This approach focuses on OctopuSV’s standardization rather than caller performance. BND conversion patterns were analyzed across SV types for each caller and platform.

To validate biological relevance, selected corrected BNDs from Manta (NA12878 NGS) were confirmed using Truvari (v4.2.2) (English *et al.* 2022), annotated by AnnotSV (v3.4.4) with parameters “-SVminSize 0 -annotationMode full -overwrite 1”, and manually inspected in IGV to verify read support and gene disruption. To evaluate potential false positives introduced by BND correction beyond the matched accuracy assessment, we compared caller outputs before and after conversion across all datasets using identical Truvari parameters.

### 2.8 Benchmarking of SV merging function

We benchmarked the merging performance of OctopuSV using the same six datasets. Comparisons were made against four widely used SV merging tools—SURVIVOR (v1.07), Jasmine (v1.1.5), SVmerge (v0.36), and CombiSV (v2.3)—across multiple merging strategies: common operations (intersection, union, minimum support thresholds) and OctopuSV-specific strategies (maximum support, caller-specific extraction, and custom set operations). Short-read SVs were called using Manta, Lumpy, SvABA, and DELLY; long-read SVs were obtained via CuteSV, PBSV, Sniffles, SVIM, SVDSS, and DeBreak. OctopuSV merging used the default parameters of its “merge” subcommand. Notably, BND correction is an integral component of OctopuSV’s workflow and was applied by default, while other tools processed original VCF files. Comparator tools were configured as follows: SURVIVOR with “1000 1 1 1 0 30,” Jasmine with “—normalize_type” and four threads, while SVmerge and CombiSV were run with default settings.

For NGS datasets, merging strategies included intersection, union, minimum support (≥2/≥3 callers), maximum support (≤2 callers), Manta-specific extraction (chosen for its clinical use), and custom set operations defined as [(Manta AND SvABA) NOT (LUMPY OR DELLY)], reflecting the different methodologies of Manta (Chen *et al.* 2016)/SvABA (Wala *et al.* 2018) (local assembly) versus LUMPY (Layer *et al.* 2014)/DELLY (Rausch *et al.* 2012) (read signals). For long-read datasets, additional minimum support levels (≥2 to ≥5 callers), Sniffles-specific extraction (Smolka *et al.* 2024), and custom set operations [(DeBreak AND SVDSS) NOT Others] were evaluated, based on shared POA methodology of DeBreak (Chen *et al.* 2023) and SVDSS (Denti *et al.* 2023). For CombiSV compatibility, an additional “4callers” union analysis (Sniffles, PBSV, CuteSV, SVIM) was performed across all datasets.

To evaluate the BND correction contribution, additional analysis used OctopuSV’s BND-corrected output as input for all tools. OctopuSV output files (SVCF format) were converted to standard VCF using the “svcf2vcf” subcommand. Performance metrics were calculated using Truvari (v4.2.2) with default parameters (position matching: 50–500 bp, size similarity: ≥0.7, --pctseq 0). For more details about SVCF, see the OctopuSV documentation (https://github.com/ylab-hi/OctopuSV).

### 2.9 Assessment of SV type consistency across merging tools

To evaluate SV type consistency during merging, we assessed type consistency across five merging tools (OctopuSV, SURVIVOR, Jasmine, SVmerge, and CombiSV). We identified variants where primary SVTYPE annotation was inconsistent with their constituent original variant types and quantified incorrectly merged variants per tool-dataset combination. Misclassification patterns were visualized using Sankey diagrams. We calculated proportions of each variant type from true-positive calls and compared them to ground truth distributions using normalized stacked bar plots.

### 2.10 Truth-free trio evaluation using Mendelian inheritance

To address potential ground truth limitations and demonstrate multi-sample capabilities, we implemented truth-free evaluation using Mendelian inheritance principles in the HG002 trio family. High-quality SV calls were obtained from the Genome in a Bottle (GIAB) consortium for each trio member: HG002 (child), HG003 (father), and HG004 (mother), generated using PBSV on PacBio HiFi data. These pre-computed VCF files were then processed through merging using OctopuSV, Jasmine (v1.1.5), SURVIVOR (v1.07), and SVmerge (v0.36) with their respective default settings. Quality filtering retained variants with SVLEN ≥30bp, except TRA and BND variants. Mendelian Violation Rate (MVR) was calculated as the proportion of variants present in child but absent in both parents (Kirsche *et al.* 2023). Analysis excluded variants in repetitive regions and low mapping quality.

### 2.11 Comparison TentacleSV with VISTA integrated framework

To position TentacleSV among integrated frameworks, we compared performance with VISTA (Sarwal *et al.* 2024) across three NGS datasets using identical callers [Manta, LUMPY, DELLY, CLEVER (Marschall *et al.* 2012)]. Since VISTA’s original implementation enforced specific caller combinations including deprecated tools, we modified input validation to accommodate our caller set while preserving its core algorithm. TentacleSV used its default consensus-based strategy (minimum support ≥2 callers), while VISTA used length-stratified approach assigning callers to specific size ranges. Performance evaluation followed the protocol described in section 2.8.

## 3 Results

### 3.1 Overview of OctopuSV architecture

**Figure 1.**
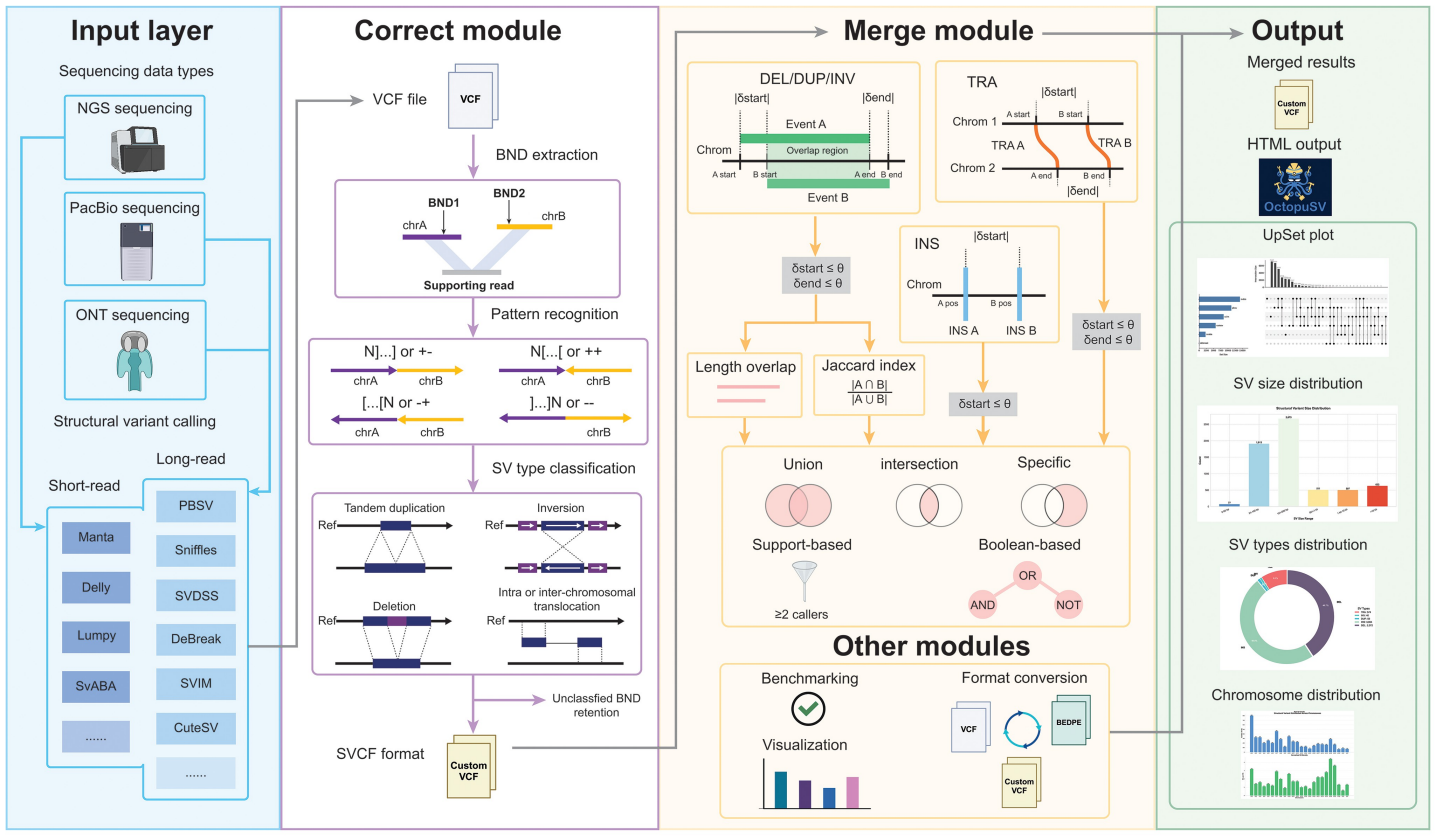
Schematic overview of OctopuSV’s architecture for structural variants standardization and merging. OctopuSV comprises three primary modules: Input layer, Correct module, and Merge module, with supporting functionality modules. The Input layer processes SV data from multiple platforms (NGS, PacBio, ONT) and handles VCF files from standard callers. The Correct module processes VCF files through BND extraction, pattern recognition of breakpoint orientations (N[.] and [.]N patterns), and SV type classification into standard forms (duplication, inversion, translocations). The Merge module implements advanced merging strategies based on event coordinates and properties, supporting various operations including length overlap assessment, Jaccard index calculation, and set operations (union, intersection, specific). This module handles different SV types (DEL/DUP/INV, TRA, INS) with specific coordinate matching criteria (δstart, δend thresholds). Additional modules provide benchmarking, format conversion, and visualization capabilities. The output includes merged results in a customized VCF format and comprehensive visualization options. The customized VCF format serves as an intermediate representation that facilitates integration between modules. Additionally, OctopuSV generates interactive HTML outputs for data exploration. Some elements were created with BioRender.com.

OctopuSV addresses two key challenges in SV analysis: standardizing ambiguous breakend annotations and integrating multi-caller, multi-sample outputs. As shown in Fig. 1, the software features three primary modules—input processing, BND correction, and SV merging—with additional functionalities for benchmarking, visualization, and format conversion. The input layer handles VCF files from multiple platforms (NGS, PacBio, ONT) and common SV callers, e.g. Manta, LUMPY, SvABA, DELLY (Rausch *et al.* 2012), PBSV, SVIM, Sniffles, CuteSV (Jiang *et al.* 2020), SVDSS (Denti *et al.* 2023), and DeBreak (Chen *et al.* 2023). The BND correction module transforms BND events into canonical SV types, while the merging module integrates outputs using intersection, union, support-based thresholds, custom set operations, and single-caller extractions. Additional features generate metrics and visualizations, with an HTML reporting system (Fig. 1, available as supplementary data at *Bioinformatics* online).

**Figure 2.**
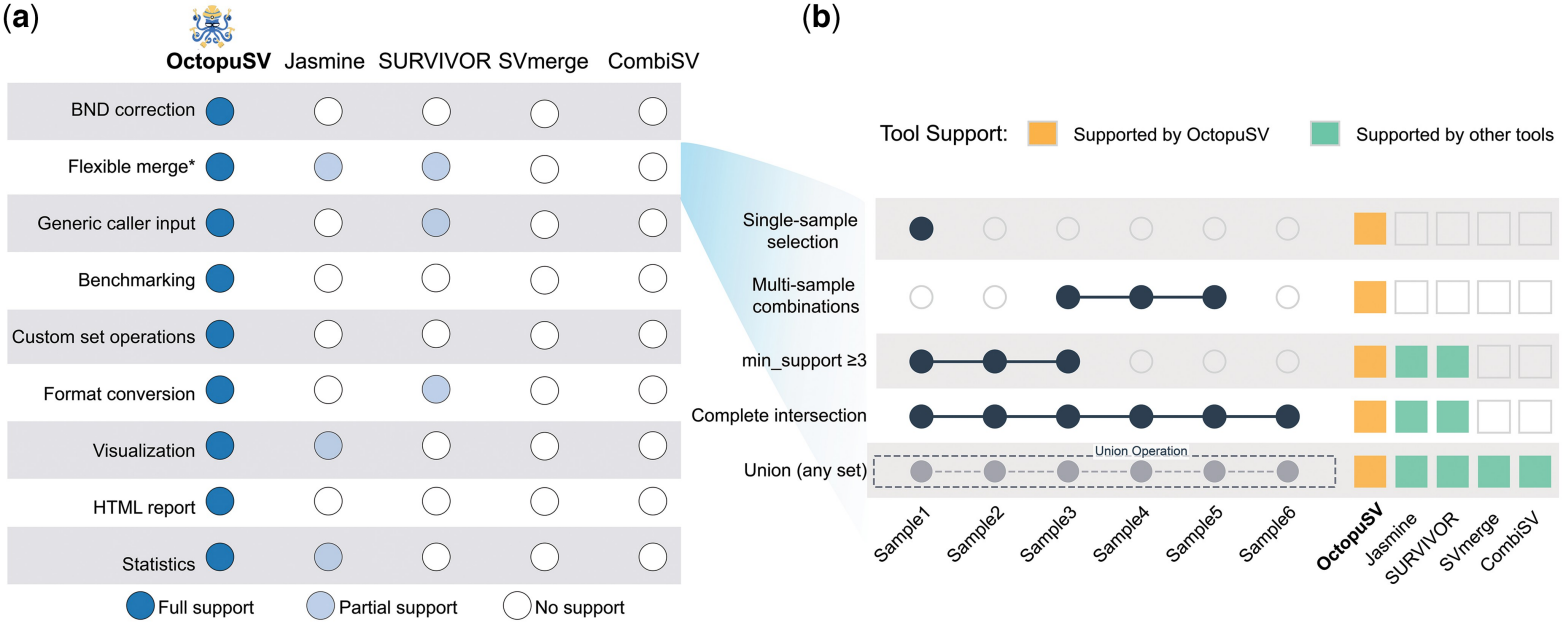
Comparison of key features between OctopuSV and existing structural variants (SVs) merging tools (Jasmine, SURVIVOR, SVmerge, and CombiSV). (a) Feature comparison matrix showing support levels (full, partial, or none) for key functionalities across tools. (b) OctopuSV supports a broader range of sample set operations, including both standard filters (e.g. minimum support, intersection, union) and more flexible options such as single-sample and subset-specific selection.

TentacleSV streamlines the entire process from raw data to final variants (Fig. 2, available as supplementary data at *Bioinformatics* online), coordinating read mapping, variant detection, and OctopuSV operations through a configuration file. This enhances reproducibility across analyses (Kosugi *et al.* 2019, Sarwal *et al.* 2024).

### 3.2 Feature comparison with existing tools

To contextualize OctopuSV’s capabilities, we compared features against four SV merging solutions: Jasmine, SURVIVOR, SVmerge, and CombiSV (Fig. 2a). While several tools, such as Jasmine and SURVIVOR, offer partial support for flexible merging or format conversion, OctopuSV provides an integrated solution that includes native BND correction, benchmarking, and custom set operations. Figure 2b showcases OctopuSV’s advanced set operation capabilities, enabling custom operations for precise variant filtering across multiple samples—functionality not available in other tools typically restricted to basic union or intersection operations.

### 3.3 BND prevalence and correction performance

**Figure 3.**
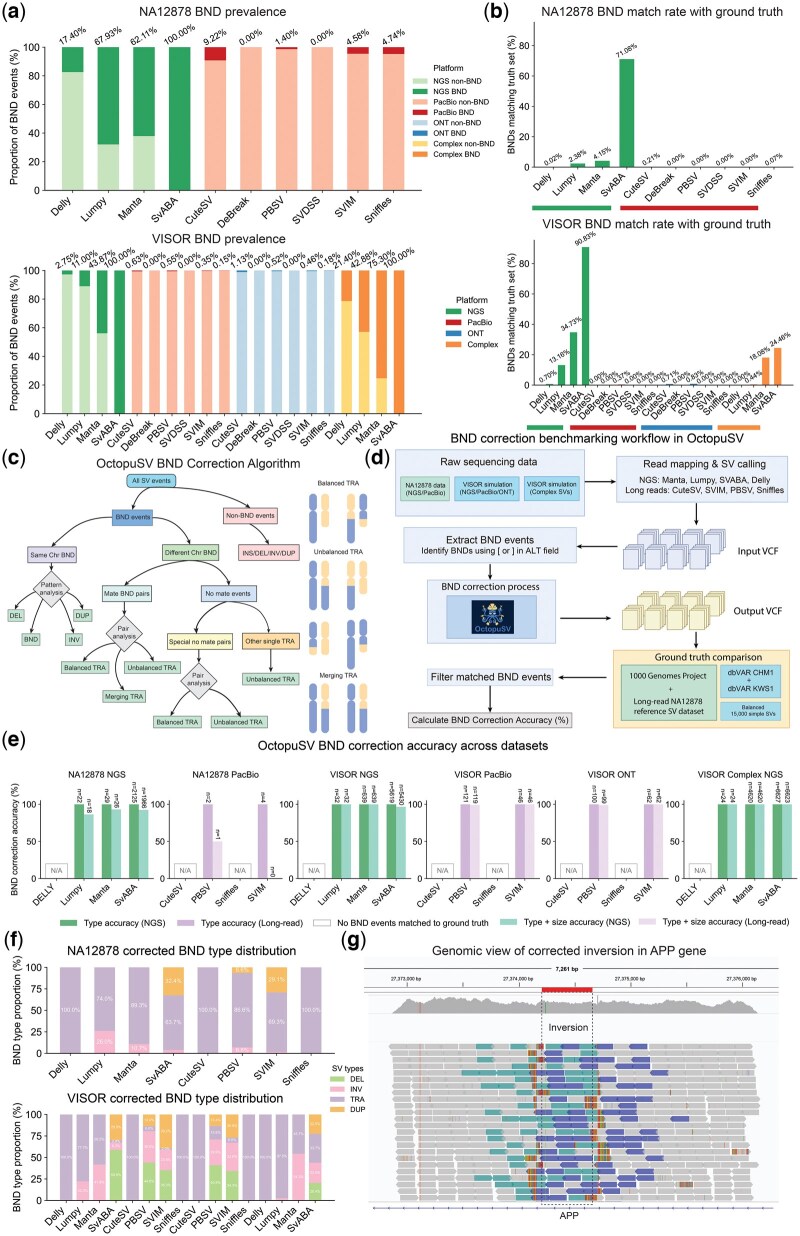
BND prevalence, correction, and benchmarking in OctopuSV. (a) Proportion of BND annotations among total structural variants reported by different callers in real (NA12878) and simulated (VISOR: NGS and complex NGS) datasets, highlighting frequent usage of BND annotations, particularly in short-read callers. (b) Proportion of BND calls matching high-confidence reference SV datasets. (c) OctopuSV’s workflow for correcting ambiguous BND annotations by classifying them into canonical SV types (DEL, INV, DUP, TRA) or selectively retaining BND format, based on chromosomal context and breakpoint patterns. The right panel illustrates translocation subtypes: balanced (reciprocal exchange), unbalanced (material gain/loss), or merging translocations (paired BND representing single events). (d) Benchmarking workflow for evaluating OctopuSV’s correction accuracy through comparison with established ground-truth datasets. (e) BND correction accuracy showing type accuracy and combined type + size accuracy for NGS and long-read callers; white boxes indicate callers without matched events. (f) Distribution of corrected BND events by canonical SV types, revealing reclassification patterns across callers and platforms. (g) Example of corrected inversion event in the APP gene from NA12878, initially identified as ambiguous BND events by Manta but resolved by OctopuSV. Some elements were created with BioRender.com.

To establish BND standardization importance, we quantified BND prevalence across callers and platforms. Analysis of NA12878 (Zook *et al.* 2019) and VISOR datasets revealed considerable variation in BND usage (Fig. 3a). In NA12878 NGS data, SvABA reported 100% of variants as BNDs, while Manta (62.11%), LUMPY (67.93%), and DELLY (17.40%) showed varying rates. Long-read callers utilized BNDs less frequently, ranging from 1.40% (PBSV) to 9.22% (CuteSV) in PacBio data. VISOR datasets exhibited similar patterns, with SvABA reporting 100% BND usage, Manta at 43.87%, and LUMPY at 11.00%. These findings confirm widespread BND annotation usage across real and simulated datasets.

We assessed BND validity against high-confidence reference sets (Fig. 3b). Match rates varied by caller: in NA12878 data, SvABA showed highest correspondence (71.08%), while other short-read callers demonstrated lower rates (Manta: 4.15%, LUMPY: 2.38%, DELLY: 0.02%). In VISOR data, SvABA again exhibited the highest match rate (90.83%), followed by Manta (34.73%) and LUMPY (13.16%). These results indicate many BND events correspond to genuine SVs requiring processing.

OctopuSV implements a hierarchical classification system (Fig. 3c) categorizing events by chromosomal context, then analyzing breakpoint patterns. We established a benchmarking framework processing BND event against ground truths (Fig. 3d), focusing solely on BNDs matching ground truth entries. OctopuSV correctly converted all matched BND notations to appropriate SV types (Fig. 3e). Combined type + size accuracy ranges from 86.4% to 100% in cases with sufficient sample sizes. To address potential false positive concerns, we compared individual caller performance before and after BND correction across all datasets. Among 24 caller-dataset combinations, false positives increased in 8 cases, decreased in 5 cases, and remained unchanged in 11 cases (Dataset 2, available as supplementary data at *Bioinformatics* online). Notably, Manta showed larger FP increases in the presence of complex variants (VISOR Complex NGS: +1984 FPs) alongside recall improvements (0.167 to 0.292), indicating that BND correction faces inherent challenges in complex genomic contexts that require careful consideration in real-world applications. The consistent conversion accuracy observed across diverse platforms and callers indicates that OctopuSV effectively standardizes BND annotations for validated SVs.

Analysis of corrected BND distributions (Fig. 3f) revealed caller-specific patterns: DELLY’s BNDs were exclusively reclassified as TRAs (100%). LUMPY’s were predominantly TRAs (73.97%) with remainder as INVs (26.03%), and Manta showed similar patterns, SvABA displayed more diversity (63.74% TRAs, 32.40% DUPs, 3.86% INVs). Long-read callers followed comparable patterns, with most showing a predominance of TRA events. These patterns were largely consistent between real and simulated datasets.

To demonstrate the biological relevance of BND correction, we identified genes containing SVs from Manta-called BND events in NA12878 that were corrected by OctopuSV and subsequently validated against reference datasets. For example, an inversion affecting *APP*, a gene implicated in Alzheimer’s disease (Jonsson *et al.* 2012), was initially reported by Manta as ambiguous BND events. However, OctopuSV correctly classified it as INV, confirmed by AnnotSV annotation and manual inspection using IGV (Fig. 3g). Other disease-associated genes were similarly identified, including a 12.5 kb inversion in the autism-associated *CNTNAP2* gene (Peñagarikano and Geschwind 2012) and a 4.8 kb inversion in *SMC1A* (linked to rare disease Cornelia de Lange syndrome) (Deardorff *et al.* 2007) (Dataset 3, available as supplementary data at *Bioinformatics* online). These results demonstrate OctopuSV’s effectiveness in standardizing BND annotations for biologically relevant SVs.

### 3.4 Evaluation of OctopuSV merging performance across diverse datasets

**Figure 4.**
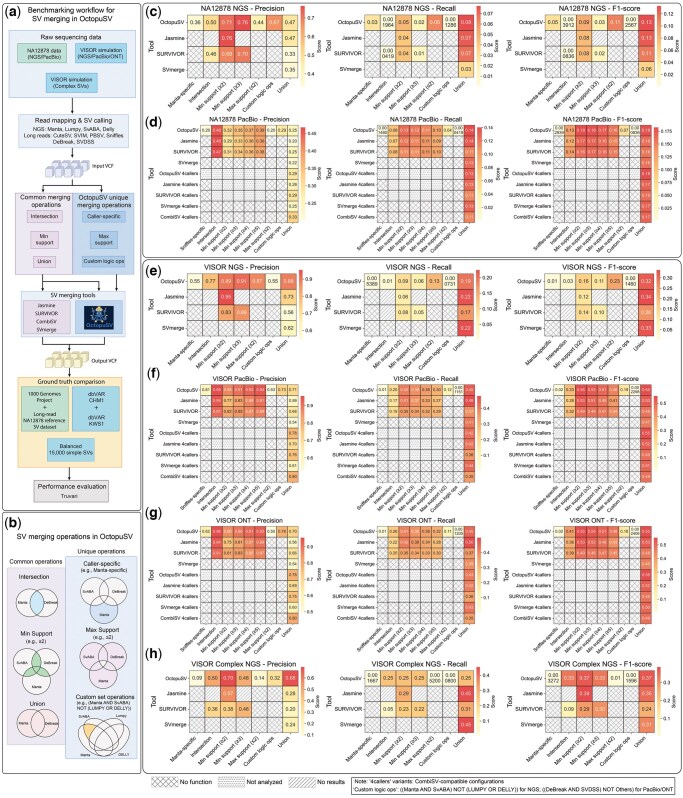
Evaluation of OctopuSV’s SV merging strategies and performance. (a) Workflow for benchmarking SV merging from raw data to performance evaluation. (b) SV merging operations supported by OctopuSV. Common operations include intersection, union, and minimum support (≥2 callers). Unique operations include maximum support (≤2 callers), caller-specific extraction, and custom set operations. For NGS datasets, custom operation is [(Manta AND SvABA) NOT (LUMPY OR DELLY)]; for PacBio/ONT datasets [(DeBreak AND SVDSS) NOT Others]. (c–h) Heatmaps comparing Precision, Recall, and F1 scores across datasets: (c) NA12878 NGS, (d) NA12878 PacBio, (e) VISOR NGS, (f) VISOR PacBio, (g) VISOR ONT, (h) VISOR Complex NGS. VISOR Complex NGS represents balanced simulation with overlaid complex variants. Cells marked with horizontal lines indicate unsupported operations; diagonal lines represent analyses with no results; dotted patterns indicate configurations not analyzed. The label “4callers” indicates analyses limited to the caller combination (Sniffles, PBSV, CuteSV, SVIM) specifically supported by CombiSV.

We compared OctopuSV’s merging performance with four established tools—Jasmine, SURVIVOR, SVmerge, and CombiSV—using real (NA12878 NGS and PacBio) and simulated (VISOR NGS, PacBio, ONT, and Complex NGS) datasets (Fig. 4a; Dataset 4, available as supplementary data at *Bioinformatics* online). Performance metrics, including precision, recall, and F1-score, were assessed under common strategies (intersection, union, minimum support thresholds) and OctopuSV-specific operations (maximum support, caller-specific extraction, and custom set operations) (Fig. 4b).

In the NA12878 NGS dataset (Fig. 4c), OctopuSV consistently outperformed other tools across merging strategies. For the intersection strategy, it achieved the highest precision (0.50) and recall (0.002), slightly exceeding SURVIVOR (0.46, <0.001), while Jasmine failed to return results. Under minimum support thresholds, OctopuSV reached precision values of 0.71 (≥2 callers) and 0.76 (≥3 callers), matching or surpassing Jasmine (0.76 at ≥2) and SURVIVOR (0.68–0.70). However, recall remained low across tools (OctopuSV: 0.047; Jasmine: 0.041; SURVIVOR: 0.042 at ≥2). The union strategy yielded improved recall (0.077), higher than Jasmine (0.073), SURVIVOR (0.068), and SVmerge (0.033).

In the NA12878 PacBio dataset (Fig. 4d), OctopuSV again demonstrated robust performance. Intersection yielded 0.46 precision and 0.077 recall, comparable to SURVIVOR (0.47, 0.080). With increasing minimum support (≥2 to ≥5), OctopuSV precision rose from 0.32 to 0.39, with stable recall (∼0.10), matching Jasmine and SURVIVOR. Under the union strategy, OctopuSV achieved the highest F1-score (0.18), outperforming Jasmine, SURVIVOR, and SVmerge (all 0.16). In the 4-caller subset (Sniffles, PBSV, CuteSV, SVIM), OctopuSV retained top performance (F1: 0.18 versus CombiSV: 0.17).

On VISOR NGS (Fig. 4e), OctopuSV uniquely returned valid intersection results (precision: 0.77, recall: 0.01), while Jasmine and SURVIVOR did not. At higher support thresholds (≥2–3), OctopuSV reached precision of 0.89–0.91 and recall of 0.06, outperforming Jasmine (precision up to 0.95, same recall) and SVmerge (0.86, 0.05). Union strategy yielded an F1-score of 0.32, on par with Jasmine (0.34) and SVmerge (0.33). VISOR PacBio (Fig. 4f) showed high precision (0.95) and recall (0.20) in intersection mode; precision peaked at 0.94 (≥5 callers) and recall at 0.30. Union F1-score was 0.55 (precision 0.71, recall 0.45), exceeding Jasmine (0.53) and SVmerge (0.46). VISOR ONT (Fig. 4g) yielded similar results: intersection precision 0.96, recall 0.26; minimum support (≥5) precision 0.93, recall 0.32; union F1-score 0.55 (precision 0.70, recall 0.45), again outperforming Jasmine (0.53) and SVmerge (0.46). VISOR Complex NGS (Fig. 4h), designed with balanced canonical SV types and overlaid complex variants, showed OctopuSV intersection precision of 0.50 and recall of 0.25 (F1: 0.33), with minimum support (≥2) precision of 0.70, recall 0.25, and F1-score 0.37. Union strategy achieved precision 0.68, recall 0.25, and F1-score 0.37, outperforming Jasmine (F1: 0.35), SURVIVOR (F1: 0.24), and SVmerge (F1: 0.31). While OctopuSV yielded lower recall under union mode, it consistently maintained a favorable balance between precision and overall F1-score. Across VISOR datasets, OctopuSV consistently outperformed CombiSV in the 4-caller union analysis (F1: 0.55 versus 0.49).

Low recall values were consistent across all tools, as seen in examples like 0.077 in NA12878 PacBio and 0.01 in VISOR NGS. This is likely due to variability in caller outputs and strict matching criteria. Despite this, OctopuSV’s advanced operations offered flexibility. Maximum support (≤2 callers) reached precision of 0.87 in VISOR NGS, caller-specific extraction (e.g. Manta for NGS, Sniffles for long-read) hit 0.62 in VISOR ONT, and custom set operations, e.g. [(DeBreak AND SVDSS) NOT Others], achieved 0.78 in VISOR ONT. These specialized merges help researchers focus on selected variants—such as highly confident ones or those found by specific callers—enabling more targeted analyses.

To evaluate the individual contribution of BND correction, we performed additional analysis where all merging tools used OctopuSV’s BND-corrected input (Figs 3 and 4, available as supplementary data at *Bioinformatics* online; Dataset 5, available as supplementary data at *Bioinformatics* online). Other tools showed improved performance with BND preprocessing: Jasmine’s union F1-scores increased (e.g. VISOR Complex NGS: 0.35–0.38), and SURVIVOR demonstrated gains across multiple scenarios (e.g. VISOR Complex NGS intersection F1: 0.09–0.29). Despite these improvements, OctopuSV maintained competitive performance in most configurations, validating both the utility of BND correction and the effectiveness of integrated design.

### 3.5 SV type consistency during merging

**Figure 5.**
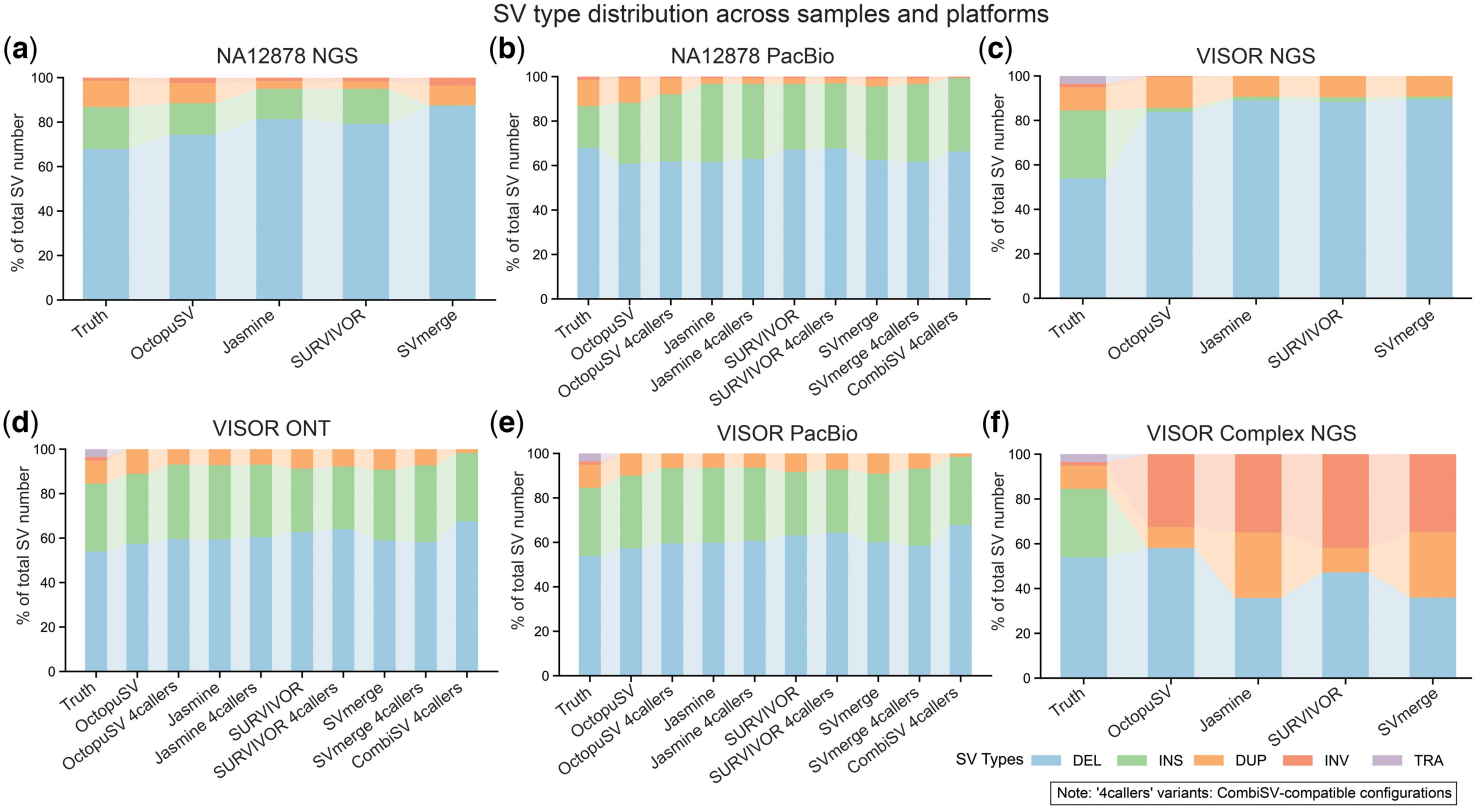
Preservation of SV type distribution for true positive SVs across merging tools and datasets. Normalized stacked bar plots comparing the distributions of true positive structural variants (SVs) identified by different merging tools (OctopuSV, SURVIVOR, Jasmine, SVmerge, CombiSV) across six datasets: (a) NA12878 NGS, (b) NA12878 PacBio, (c) VISOR NGS, (d) VISOR ONT, (e) VISOR PacBio, and (f) VISOR Complex NGS. Each subplot specifically compares the SV type distribution of correctly identified variants (true positives) obtained by each tool with the ground truth distribution, illustrating each merging tool’s fidelity and consistency in preserving the original SV type annotations.

We evaluated whether merging tools introduce systematic type misclassifications across five merging tools—OctopuSV, SURVIVOR, Jasmine, SVmerge, and CombiSV—using the same datasets as in the merging performance analysis (NA12878 NGS, NA12878 PacBio, VISOR NGS, VISOR ONT, VISOR PacBio, and VISOR Complex NGS; Fig. 5). This analysis revealed distinct differences in variant type fidelity during merging.

**Figure 6.**
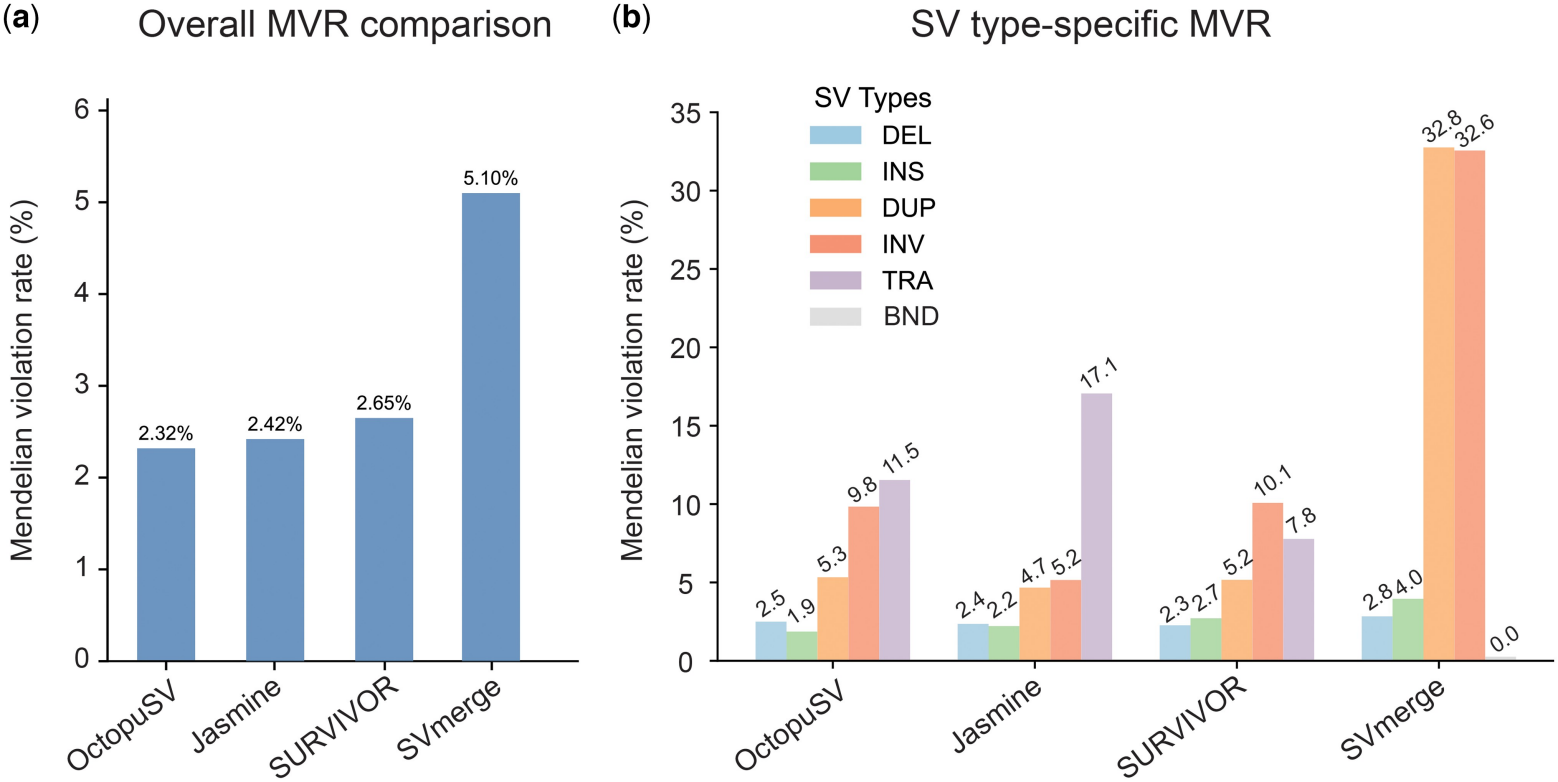
Truth-free trio evaluation of SV merging tools using Mendelian inheritance. (a) Overall Mendelian Violation Rate (MVR) comparison across four tools using HG002 trio analysis. Lower MVR indicates fewer false positives. (b) SV type-specific MVR breakdown across categories (DEL, INS, DUP, INV, TRA, BND). Analysis used high-quality SV calls from GIAB for HG002 trio (child, father, mother) using PBSV on PacBio HiFi data. MVR calculated as proportion of variants present in child but absent in both parents.

**Figure 7.**
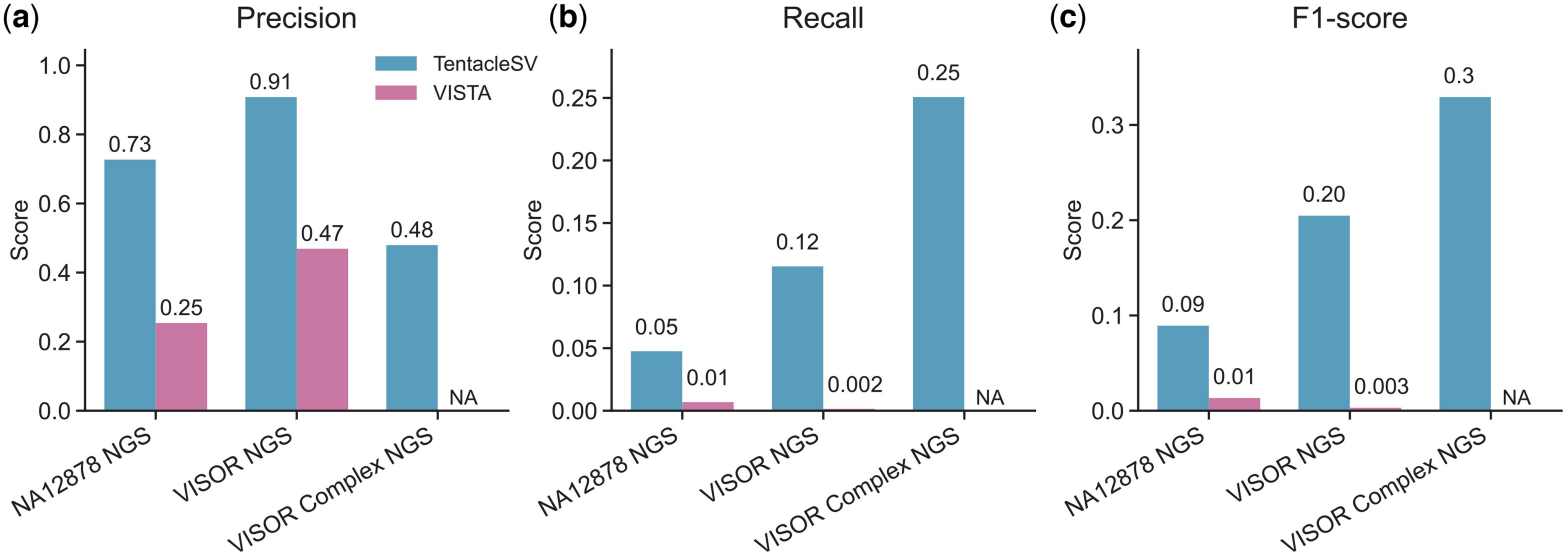
Integrated SV analysis framework comparison. Performance comparison between TentacleSV and VISTA across three NGS datasets: (a) precision, (b) recall, (c) F1-score. TentacleSV uses consensus-based merging (≥2 callers), VISTA uses length-stratified assignment. Both used identical callers (Manta, LUMPY, DELLY, CLEVER). VISTA failed to process VISOR Complex NGS (marked as NA).

SURVIVOR showed substantial inconsistencies, with incorrect merges ranging from 183 instances in VISOR PacBio to 2477 in VISOR NGS data (Fig. 5, available as supplementary data at *Bioinformatics* online). These errors involved mismatches where the primary SVTYPE annotation differed from the original types of constituent variants, such as merging INV into DEL or DUP into TRA. Sankey diagrams illustrated these SV type annotation changes (Figs 6 and 7, available as supplementary data at *Bioinformatics* online), showing frequently observed SV type reclassifications (e.g. INV to DEL, DEL to INV) in SURVIVOR’s outputs. This included 935 INV-to-DEL misclassifications in NA12878 NGS (Fig. 6a, available as supplementary data at *Bioinformatics* online) and 2004 DEL-to-INV conversions in VISOR NGS (Fig. 6c, available as supplementary data at *Bioinformatics* online). In contrast, OctopuSV, Jasmine, SVmerge, and CombiSV reported zero incorrect merges across all datasets.

We also assessed whether each tool introduces type misclassifications, focusing on true positive calls in union mode (Figs 5a–f). In NA12878 NGS (Fig. 5), OctopuSV’s detected variants comprised DEL at 79.5%, INS at 14.8%, and DUP at 4.0% (ground truth: DEL 67.9%, INS 18.9%, DUP 11.8%). Other tools showed similar patterns with DEL enrichment, such as Jasmine (DEL 81.3%, INS 13.7%, DUP 3.5%) and SURVIVOR (DEL 79.7%, INS 15.3%, DUP 3.3%). In NA12878 PacBio (Fig. 5b), OctopuSV maintained DEL at 64.2%, INS at 32.4%, and DUP at 2.7%.

In simulated datasets, platform-specific detection patterns emerged. For VISOR NGS (Fig. 5c), all merging tools showed substantial INS reduction: OctopuSV’s callset contained 2.0% INS variants (ground truth: 30.6%), with similar limitations observed across tools (Jasmine 1.7%, SURVIVOR 2.0%). This contrasts with NA12878 NGS results where INS recovery was more successful (OctopuSV 14.8%), This reflects the fundamental challenge that short-read callers face with novel sequence insertions compared to mobile elements and reference-based duplications found in real genomes (Gong *et al.* 2021). Long-read platforms demonstrated improved INS recovery: VISOR ONT (Fig. 5d) showed OctopuSV INS at 31.6% versus ground truth 30.6%, while VISOR PacBio (Fig. 5e) achieved 31.1% INS recovery. The VISOR Complex NGS dataset (Fig. 5f) revealed extreme challenges, with complete INS loss across all tools and substantial INV enrichment (OctopuSV 32.6%, Jasmine 35.0%, SURVIVOR 41.8%), confirming that short-read technologies struggle particularly with novel sequence insertions in complex genomic contexts.

### 3.6 Truth-free trio evaluation validates OctopuSV’s multi-sample merging capabilities

We evaluated multi-sample merging performance using truth-free Mendelian inheritance analysis across the HG002 trio family. Analysis of 45 000+ structural variants revealed OctopuSV achieved the lowest overall MVR of 2.32%, compared to Jasmine (2.42%), SURVIVOR (2.65%), and SVmerge (5.10%) (Fig. 6a). These results are comparable to high-quality variant calling tools in trio analysis (Lin *et al.* 2022).

SV type-specific analysis (Fig. 6b) showed that OctopuSV achieved 2.5% MVR for DEL and 1.9% for INS, matching or exceeding other tools. OctopuSV demonstrated 5.3% MVR for duplications and 9.8% for inversions, compared to higher SVmerge rates (32.8% duplications, 32.6% inversions). For TRA, OctopuSV maintained 11.5% MVR versus Jasmine’s 17.1%. This demonstrates OctopuSV’s effectiveness in multi-sample scenarios without dependence on potentially incomplete reference datasets.

### 3.7 Integrated SV analysis framework comparison

We compared TentacleSV against VISTA, another integrated framework, across three NGS datasets (Fig. 7). TentacleSV consistently outperformed VISTA across metrics. On NA12878 NGS, TentacleSV achieved precision 0.73 versus 0.25, recall 0.05 versus 0.01, and F1-score 0.09 versus 0.01. VISOR NGS showed similar trends with TentacleSV reaching precision 0.91 versus 0.47, recall 0.12 versus 0.002, and F1-score 0.20 versus 0.003. On VISOR Complex NGS, TentacleSV maintained balanced performance (precision 0.48, recall 0.25, F1-score 0.33), while VISTA failed to produce valid results for this challenging dataset.

## 4. Discussion and conclusion

Accurate SV detection and integration across diverse samples, callers, and platforms remain challenging for genomic research and clinical diagnostics. We introduced OctopuSV and TentacleSV to address three key issues: (i) standardizing ambiguous BND annotations, (ii) enabling flexible SV merging strategies, and (iii) automating SV analysis workflows. By systematically converting BNDs into canonical SV types (DEL, INV, DUP, TRA), OctopuSV recovers biologically important variants otherwise discarded as ambiguous breakpoints. Benchmarking demonstrated that these conversions significantly enhance interpretability and consistency in multi-caller or multi-sample analyses.

OctopuSV’s effectiveness stems from its integrated design combining BND correction with advanced merging capabilities. Additional analysis where other tools used our BND correction confirmed that while BND preprocessing improves performance across tools, OctopuSV maintains competitive results through co-optimized algorithms. Beyond BND standardization, OctopuSV’s flexible merging capabilities include advanced set operations such as difference, complement, and user-defined merges. Basic merging approaches (intersection, union, minimum support) are commonly used, but the inclusion of these advanced operations enables precise extraction of SV subsets. In clinical settings, such operations facilitate detection of tumor-specific variants relative to controls. Technical applications, such as caller-specific extractions [e.g. (Manta AND SvABA) NOT (LUMPY OR DELLY)], guide SV caller selection and pipeline optimization. Similarly, OctopuSV’s maximum support feature (variants supported by ≤2 callers) enables exploration of rare variants, potentially differentiating biological events from artifacts. Importantly, OctopuSV preserves SV type fidelity during merging, overcoming misclassification errors in some merging tool.

We also conducted comprehensive evaluations to validate OctopuSV’s performance across diverse scenarios. Using balanced simulation with canonical SV types and overlaid complex variants, OctopuSV maintained stable performance in challenging genomic contexts with relatively high F1-scores and higher precision compared to alternative strategies. While recall performance faces challenges when simple and complex variants coexist, this reflects real-world difficulties in SV detection within structurally complex regions affecting all methods. Given challenges in obtaining comprehensive ground truth datasets, we used truth-free trio evaluation using Mendelian inheritance principles for orthogonal validation. This approach showed OctopuSV performed favorably compared to competing tools without relying on potentially incomplete reference datasets, establishing its utility in multi-sample analyses.

By integrating these functionalities into the Snakemake-based TentacleSV pipeline, we developed a comprehensive framework for end-to-end SV analysis. Compared with VISTA, TentacleSV consistently achieved better performance across datasets, outperforming VISTA’s length-stratified approach and successfully handling complex SVs that VISTA failed to process (Liu *et al.* 2022).

Despite these advantages, OctopuSV has limitations. First, its accuracy depends on upstream SV callers, inheriting their biases and errors (Tian *et al.* 2016). Consequently, merged callset distributions reflect these caller biases and low recall rather than unbiased sampling, but merging tool choice critically impacts type annotation fidelity. Second, detecting SVs in complex regions (e.g. centromeres, telomeres, gaps) remains difficult, often yielding inconsistent results across tools (Dierckxsens *et al.* 2021, Wang *et al.* 2025). Our BND correction analysis shows controlled false positive rates in most settings, but complex regions still challenge precision-recall balance. Likewise, overlapping SVs reduce recall in complex contexts, underscoring a broader limitation in handling complex rearrangements. Incorporating repeat-aware filters or confidence-scoring schemes may improve robustness. Third, benchmarking is limited by available reference datasets. The NA12878 ground truth dataset contains 73 287 SVs with inflated deletion proportions (67.9%), substantially exceeding the 20 000–30 000 SVs typically expected in single genomes. This contributes to observed low recall rates (e.g. 0.14). However, part of the reduction likely reflects limitations inherited from upstream callers and challenges in complex genomic regions, as discussed above. Our detection of ∼10 000–11 000 variants likely represents realistic single-genome estimates rather than methodological failure. Future evaluations require reference datasets that better reflect physiological SV distributions and quantities. Lastly, while our custom set operations are useful, further clinical validation is needed for translational use.

OctopuSV and TentacleSV lower barriers between experimental and computational users, supporting tasks from tumor SV discovery to rare disease variant detection. Future directions include integrating functional annotations, variant prioritization, and annotation-based filtering. We also envision leveraging machine learning (Vadapalli *et al.* 2022) and pan-genomic frameworks to enhance performance and clinical utility.

## Supplementary Material

btaf599_Supplementary_Data

## Data Availability

The OctopuSV and TentacleSV software packages are freely available at GitHub (https://github.com/ylab-hi/OctopuSV; https://github.com/ylab-hi/TentacleSV) under the MIT license. A versioned archive of OctopuSV is also available via Zenodo (https://doi.org/10.5281/zenodo.16618917). All analysis scripts, configurations, and benchmarking results used in this paper are available at GitHub (https://github.com/qingxiangguo/OctopuSV_paper). The GRCh38 reference genome was obtained from GENCODE (https://ftp.ebi.ac.uk/pub/databases/gencode/Gencode_human/release_38/GRCh38.p13.genome.fa.gz). For real data evaluation, we used the NA12878 NIST HG001 HiSeq subsampled 30× data (ftp://ftp-trace.ncbi.nlm.nih.gov/giab/ftp/data/NA12878/NIST_NA12878_HG001_HiSeq_300x) and PacBio SequelII CCS data from GIAB (https://ftp-trace.ncbi.nlm.nih.gov/giab/ftp/data/NA12878/PacBio_SequelII_CCS_11kb/HG001.SequelII.pbmm2.hs37d5.whatshap.haplotag.RTG.trio.bam). The ground truth sets used for read-data benchmarking include variants from the 1000 Genomes Project (ftp://ftp.1000genomes.ebi.ac.uk/vol1/ftp/phase3/integrated_sv_map/ALL.wgs.mergedSV.v8.20130502.svs.genotypes.vcf.gz) and the long-read assembly based NA12878 reference SV dataset (https://github.com/stat-lab/EvalSVcallers/blob/master/Ref_SV/NA12878_DGV-2016_LR-assembly.vcf) (Kosugi *et al.* 2019). The simulated SV callsets were generated using variants from dbVAR, including CHM1 (ftp://ftp.ncbi.nlm.nih.gov/pub/dbVar/data/Homo_sapiens/by_study/vcf/nstd137.GRCh37.variant_call.vcf.gz) and KWS1 (https://ftp.ncbi.nlm.nih.gov/pub/dbVar/data/Homo_sapiens/by_study/vcf/nstd137.GRCh38.variant_call.vcf.gz) datasets. The high-quality SV calls are generated from AshkenazimTrio PacBio HiFi data (https://ftp.ncbi.nih.gov/giab/ftp/data/AshkenazimTrio/analysis/PacBio_HiFi-Revio_20231031/).
