# Supplementary material for: OctopuSV and TentacleSV: a one-stop toolkit for multi-sample, cross-platform structural variant comparison and analysis
Source: Bioinformatics. 2025 Oct 31;41(11):btaf599. doi: 10.1093/bioinformatics/btaf599 (PMC12646641; doi:10.1093/bioinformatics/btaf599)
Supplement: btaf599_Supplementary_Data [file btaf599_supplementary_data.zip › supplementry_files_and_data/Supplementary_infomation_v4.docx]

**Supplementary Information for**

**OctopuSV and TentacleSV: a one-stop toolkit for multi-sample, cross-platform structural variant comparison and analysis**

Qingxiang Guo^1†*^, Yangyang Li^1†^, Tingyou Wang^1^, Abhi Ramakrishnan^1^, Rendong Yang^1,2*^

^1^Department of Urology, Northwestern University Feinberg School of Medicine, 303 E Superior St, Chicago, 60611, IL, USA.

^2^Robert H. Lurie Comprehensive Cancer Center, Northwestern University Feinberg School of Medicine, 675 N St Clair St, Chicago, 60611, IL, USA.

*Corresponding authors. E-mail(s): rendong.yang@northwestern.edu; qingxiang.guo@northwestern.edu

Contributing authors: qingxiang.guo@northwestern.edu; yangyang.li@northwestern.edu; tywang@northwestern.edu; abhirami.ramakrishnan@northwestern.edu;

† These authors contributed equally to this work.

**Supplementary Figure**

**Supplementary Figure 1.** **HTML report from OctopuSV's statistical analysis module.** This figure displays the key visualizations generated by OctopuSV's reporting system. Panels show essential structural variant metrics: **a-b** sample information, **c** genotype frequencies, **d, h** variant type composition, **e, i** size distributions, **f** quality metrics, and **g** chromosomal distribution. The HTML format allows researchers to interact with the data for efficient exploration of complex SV profiles.

**Supplementary Figure 2.** **Workflow diagram of TentacleSV pipeline.** It processes both short-read and long-read sequencing data (FASTQ/BAM) through multiple SV callers and merges the results using OctopuSV to generate a final VCF output.

**Supplementary Figure 3.** **Impact of BND correction preprocessing on SV merging tool performance.** Heatmaps comparing precision, recall, and F1-scores when all merging tools (OctopuSV, Jasmine, SURVIVOR, SVmerge) use OctopuSV's BND correction as preprocessing, followed by their respective merging algorithms across NA12878 datasets: **a** NA12878 NGS, **b** NA12878 PacBio. This analysis demonstrates that while BND correction generally improves performance across tools, OctopuSV's integrated design maintains competitive advantages. Cells marked with diagonal lines indicate unsupported operations; dotted patterns indicate configurations not analyzed. The label "4callers" indicates analyses limited to the caller combination (Sniffles, PBSV, CuteSV, SVIM) specifically supported by CombiSV.

**Supplementary Figure 4.** **Impact of BND correction preprocessing on SV merging tool performance.** Heatmaps comparing precision, recall, and F1-scores when all merging tools (OctopuSV, Jasmine, SURVIVOR, SVmerge) use OctopuSV's BND correction as preprocessing, followed by their respective merging algorithms across VISOR datasets: **a** VISOR NGS, **b** VISOR PacBio, **c** VISOR ONT, and **d** VISOR Complex NGS. This analysis demonstrates that while BND correction generally improves performance across tools, OctopuSV's integrated design maintains competitive advantages. Cells marked with diagonal lines indicate unsupported operations; dotted patterns indicate configurations not analyzed. The label "4callers" indicates analyses limited to the caller combination (Sniffles, PBSV, CuteSV, SVIM) specifically supported by CombiSV.

**Supplementary Figure 5.** **Evaluation of SV type consistency during merging across different tools.** Comparison of SV type concordance across five datasets, showing varying degrees of type preservation among different tools.

**Supplementary Figure 6.** **Sankey diagrams visualizing SV type transitions during the merging process for SURVIVOR across NA12878 datasets**: **a** NA12878 NGS, **b** NA12878 PacBio. Flow widths represent frequency of specific type transitions, with annotation boxes providing counts of each transition pattern.

**Supplementary Figure 7.** **Sankey diagrams visualizing SV type transitions during the merging process for SURVIVOR across VISOR datasets**: **a** VISOR NGS, **b** VISOR PacBio, **c** VISOR ONT, and **d** VISOR Complex. Flow widths represent frequency of specific type transitions, with annotation boxes providing counts of each transition pattern.
